# Management of Giant Bilateral Adrenal Myelolipomas in Congenital Adrenal Hyperplasia

**DOI:** 10.1210/jcemcr/luaf169

**Published:** 2025-07-31

**Authors:** Aayush Purohit, Maximilien Rappaport

**Affiliations:** University of South Carolina School of Medicine Greenville, Greenville, SC 29605, USA; Division of Endocrinology, Prisma Health Upstate Department of Medicine, Greenville, SC 29605, USA

**Keywords:** adrenal myelolipoma, congenital adrenal hyperplasia, adrenalectomy

## Abstract

Adrenal myelolipomas are rare, benign lesions containing mature adipose tissue and hematopoietic elements, typically identified incidentally on radiographic imaging. Management of small masses less than 5 cm usually requires no intervention, whereas those exceeding 6 cm in size are classified as “giant” and can cause mass effect and potentially fatal hemorrhage. Surgery must be considered for masses deemed at risk to cause complications. A 44-year-old man with a history of congenital adrenal hyperplasia presented with giant bilateral adrenal myelolipomas, which on computed tomography (CT) imaging showed significant displacement of the kidneys and compression of the inferior vena cava. Open adrenalectomy was performed, resulting in the resection of a 30.0-cm left adrenal myelolipoma and a 27.5-cm right adrenal myelolipoma. The postoperative course was uneventful. Bilateral adrenal myelolipomas of this size have rarely been reported. Management of such giant adrenal masses requires an individualized treatment approach. Bilateral adrenalectomy proved to be a safe and effective option for this patient after performing shared decision-making regarding quality of life. Highlighted here is the importance of patient-centered care in management of similar benign lesions.

## Introduction

Myelolipoma refers to a neoplasm composed of mature adipocytes and hematopoietic cells of both myeloid and erythroid lineage, first described in 1905 [1]. These lesions are often found incidentally on radiographic imaging, typically appearing as well-defined, slow-growing masses [2]. Myelolipomas are the second most common benign adrenal tumors [3].

Bilateral adrenal myelolipomas are rare, with an incidence of 0.08% to 0.4% on autopsy [4]. Lesions exceeding 15 cm in size are exceedingly uncommon, underscoring their rarity [5-7]. Larger lesions, particularly when found bilaterally, can exert mass effect, displacing organs such as the kidneys, spleen, and pancreas, and compressing blood vessels [7, 8]. This may result in symptoms such as flank pain, abdominal discomfort, hypertension, and hemorrhage in emergent situations [9-11]. Other potential symptoms include weight changes, abdominal distension, early satiety, fatigue, headaches, nausea, vomiting, and diarrhea [9, 10]. Given their size and anatomical proximity to critical structures, giant adrenal myelolipomas can necessitate open surgical resection. Here we present a patient with giant bilateral adrenal myelolipomas managed with initial observation, and subsequent surgical intervention.

## Case Presentation

A 44-year-old male individual with a history of congenital adrenal hyperplasia (CAH) due to 21-hydroxylase deficiency, and with testicular adrenal rest tumors, was referred to endocrinology after receiving a fluorodeoxyglucose (FDG)-positron emission tomography (PET) scan during treatment of a squamous cell tonsillar carcinoma. The scan revealed bilateral upper retroperitoneal heterogeneous masses. A computed tomography (CT)-guided biopsy of the left adrenal mass suggested either adrenal lipoma or myelolipoma. Considering recent burdensome therapy for tonsillar cancer, a decision was made through shared decision-making to monitor the adrenal masses without intervention. The patient was followed annually by endocrinology, with non-contrast CT imaging of the abdomen and pelvis performed 3 years after initial detection and a contrast-enhanced CT angiography (CTA) scan 4 years after initial detection. Imaging showed no significant change in mass size. After 4 years of surveillance, the patient met with endocrine surgery. He elected to proceed with bilateral adrenalectomy given significant displacement of his abdominal organs evident on CT imaging and risk of progression of disease over time.

The patient was suspected to have CAH at the age of 4 when pubarche occurred, reportedly confirmed with 24-hour urine testing. Details of his pediatric glucocorticoid treatment are uncertain, but the patient believed that he was treated with cortisone acetate 50 mg injections every other day until the age of 17, accompanied by prednisone and dexamethasone as needed. For a condition like CAH, this alternate-day regimen at such a dosage would likely have been inadequate to achieve the consistent adrenal androgen suppression required. At the age of 23, he was found to have multiple bilateral hypoechoic masses consistent with intratesticular adrenal rests on testicular ultrasound. Testicular volume on physical examination at initial presentation was 5 mL bilaterally without lesions or lumps. Medical history on presentation was also pertinent for hypothyroidism, hypogonadism (secondary to testicular rest tumors), and adrenal insufficiency. Hypothyroidism was secondary to external beam radiation therapy for squamous cell carcinoma of the tonsils.

## Diagnostic Assessment

**Figure 1.**
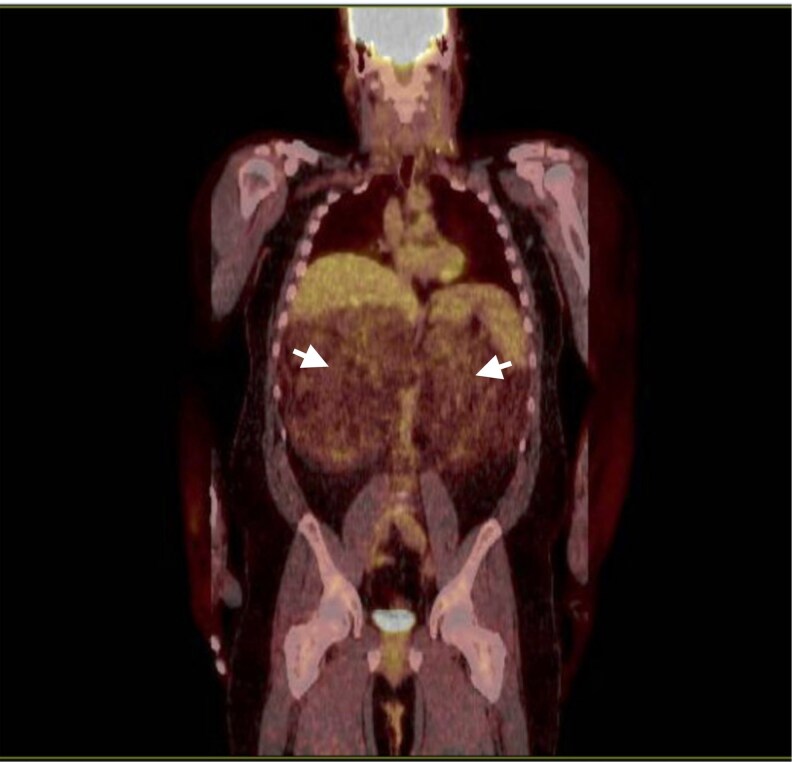
Fluorodeoxyglucose (FDG)-positron emission tomography (PET) scan coronal view. White arrows indicate adrenal masses. This scan was performed before the initial clinic visit and shows bilateral upper retroperitoneal heterogenous masses. Maximum standardized uptake value (SUV) within this retroperitoneal mass measured approximately 2.3, similar to slightly greater than background mediastinum.

**Figure 2.**
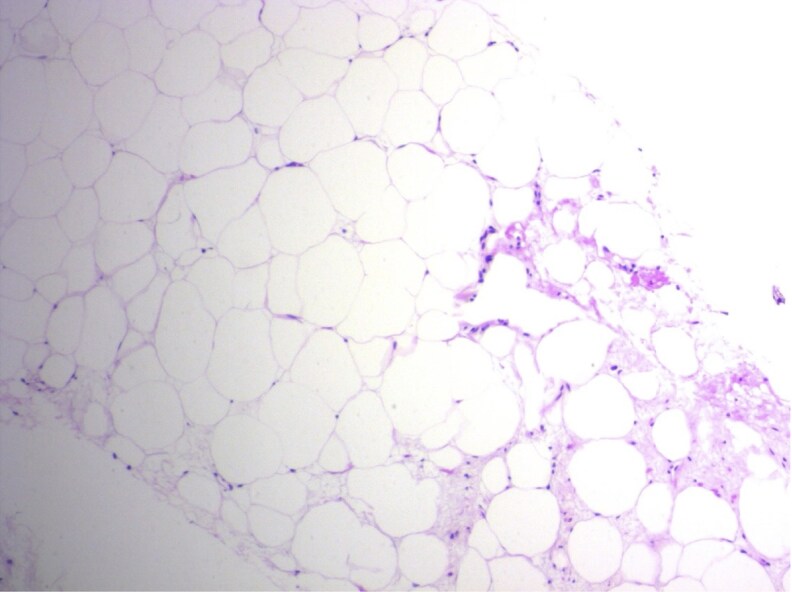
Computed tomography (CT)-guided needle biopsy of the left adrenal gland mass showing mature adipose tissue (hematoxylin and eosin; 100×).

**Figure 3.**
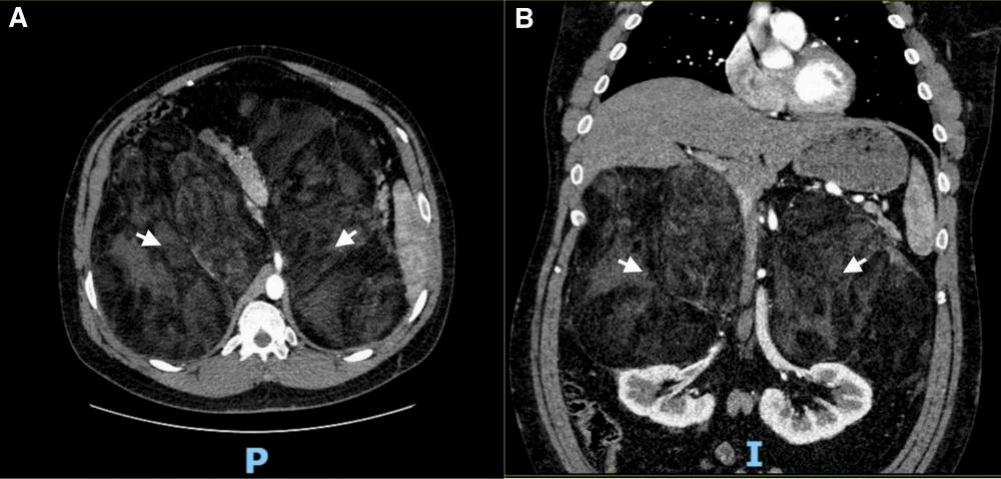
Computed tomography angiogram (CTA) abdomen with and without contrast in (A) axial view and (B) coronal view. (P) Indicating posterior side and (I) indicating inferior side. White arrows indicate adrenal masses. The right adrenal lesion measures 24.3 × 17.4 centimeters (cm). The left adrenal lesion measures 27.4 × 13.4 cm. Bilateral renal arteries effaced inferiorly secondary to mass effect from the large bilateral macroscopic fat-containing adrenal masses. The inferior vena cava is compressed by the large right retroperitoneal mass without evidence of occlusion or thrombosis. The kidneys are displaced inferiorly secondary to the adrenal masses. The unenhanced CT attenuations of the masses were −41.50 Hounsfield units (HU)for the right mass and −55.64 HU for the left mass.

Testing of serum markers was done at initial encounter, with results consistent with uncontrolled CAH (Table 1). A FDG-PET scan of the patient (Fig. 1) showed bilateral upper retroperitoneal masses likely containing fat and soft tissue components on attenuation correction images, measuring 19 × 32 cm with mass effect upon the remaining fat within the anterior upper abdomen, along with significant displacement of the duodenum and kidneys. CT-guided biopsy of the adrenal mass (Fig. 2) showed mature adipose tissue but no myelopoietic elements were present in the sampled tissue, suggesting that the masses were either lipomas or myelolipomas. Four years after initial presentation, CTA of the abdomen (Fig. 3) showed continued displacement of the duodenum and kidneys, along with effacement of bilateral renal arteries and compression of the inferior vena cava. The masses had not had significant change in size. Collectively, evidence pointed to giant adrenal myelolipomas secondary to uncontrolled stimulation of tissue in the adrenal glands.

**Table 1.** Summary of laboratory data (serum values) on initial visit for evaluation

Laboratory markers	Values (SI units)	Reference range (SI units)
Sodium	134 mEq/L (134 mmol/L)	134-144 mEq/L (134-144 mmol/L)
Potassium	**3.2 mEq/L (3.2 mmol/L)**	3.5-5.2 mEq/L (3.5-5.2 mmol/L)
Creatinine	**0.60 mg/dL (53.04 µmol/L)**	0.76-1.27 mg/dL (67-112 µmol/L)
Calcium	**7.5 mg/dL (1.9 mmol/L)**	8.7-10.2 mg/dL (2.2-2.6 mmol/L)
17-hydroxyprogesterone (17-OHP)	**3564 ng/dL (108 nmol/L)**	33-195 ng/dL (1.0-5.9 nmol/L)
Androstenedione	**317 ng/dL (11.1 nmol/L)**	40-190 ng/dL (1.4-6.6 nmol/L)
Cortisol	5.9 µg/dL (162.8 nmol/L)	4.0-20.0 µg/dL (110.4-552.0 nmol/L)
Dehydroepiandrosterone sulfate (DHEAS)	**38 µg/dL (1.0 µmol/L)**	70-495 µg/dL (1.8-12.9 µmol/L)
Aldosterone	4 ng/dL (0.11 nmol/L)	0-28 ng/dL (0.00-0.78 nmol/L)
Renin activity	**19.04 ng/mL/h (19.04 µg/L/h)**	0.25-5.82 ng/mL/h (0.25-5.82 µg/L/h)
Adrenocorticotrophic hormone (ACTH)	**66.2 pg/mL (66.2 ng/L)**	7.2-63.3 pg/mL (7.2-63.3 ng/L)
Total testosterone	**87 ng/dL (3.0 nmol/L)**	240-871 ng/dL (8.3-30.2 nmol/L)

Abnormal values bolded.

## Treatment

Preoperative planning involved shared decision-making, with the patient opting for a standard midline celiotomy as opposed to a bilateral subcostal incision. Anticipated challenges included significant blood loss due to the extensive retroperitoneal involvement and prolonged operative time. Careful dissection was required to address compression of adjacent abdominal structures, including the kidneys and the inferior vena cava.

**Figure 4.**
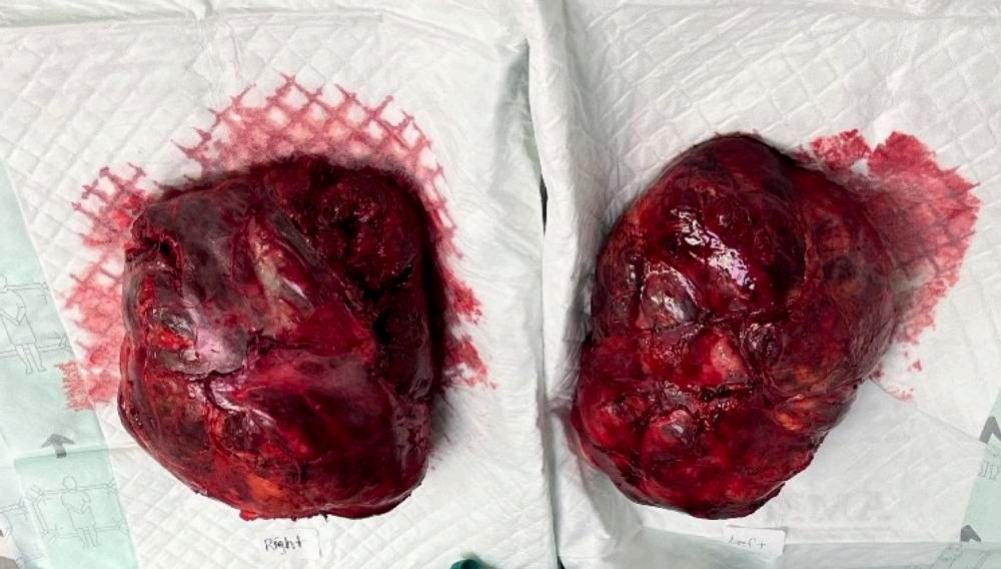
Post-adrenalectomy images of resected specimens. The left adrenal mass (right side of image) was a 3296-gram (g), 30.0 × 22.0 × 9.5-cm multinodular mass with scant surrounding fibrofatty soft tissue. Sectioning revealed lobulated cut surface with focal area of necrosis less than 1% of mass volume. No calcification, and no definitive adrenal tissue were identified. The right adrenal mass (left side of image) was a 3450-g, 27.5 × 22.5 × 11 cm multinodular mass with scant surrounding fibrofatty soft tissue and no hilar structures identified. Sectioning revealed lobulated cut surface with central cavitation filled with friable debris approximating less than 5% of mass volume. No calcifications or definitive adrenal tissue were identified.

**Figure 5.**
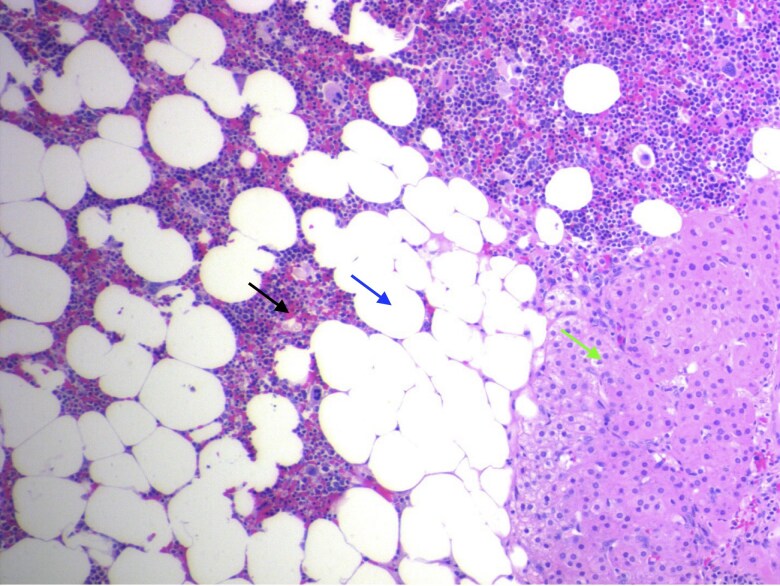
Histopathologic image of the resected mass showing hematopoiesis with supporting vascular elements (left black arrow), adipose cells (middle blue arrow), and residual adrenal cortical cells (right green arrow) (hematoxylin and eosin; 100×). Abundant lobules of well-differentiated adipocytes evident throughout, with hematopoietic cells intermixed and a lack of cellular atypia, supports benign nature of mass and confirms diagnosis of myelolipoma.

After initial evaluation, the patient was placed on hydrocortisone 20 mg in the morning and 10 mg in the evening. He was eventually brought down to 10 mg of hydrocortisone daily which was continued up until his surgery. Additionally, he was on levothyroxine 125 μg daily preoperatively, and testosterone cypionate 200 mg every 14 days. The patient underwent an open bilateral adrenalectomy. On visualization, the spleen, pancreas, and stomach were displaced cranially, and the kidney was severely displaced inferomedially. The inferior, lateral, and medial borders of the left adrenal gland were dissected from surrounding tissues, and the superior attachment of the adrenal gland was dissected from the pancreas and spleen. The left adrenal gland was then delivered (Fig. 4) with retroperitoneal bleeding noted from branches of the splenic vein. On the right side, the liver was noted to be compressed and compacted against the diaphragm. Inferior medial and lateral aspects of the right sided adrenal mass were dissected from surrounding tissue, allowing mobilization and delivery of the mass (Fig. 4). Each mass's dissection took 2 hours in duration and significant retroperitoneal bleeding was observed. Samples from the resected masses were sent for pathologic analysis (Fig. 5). Approximately 4000 mL of blood loss occurred intraoperatively, replaced with crystalloid, 4 units of packed red blood cells, 6 units fresh frozen plasma, 30 units cryoglobulin, and 1 unit of platelets. Both kidneys appeared viable and hemostasis was confirmed with no active bleeding.

## Outcome and Follow-Up

The patient was admitted to the medical intensive care unit postoperatively. He had acute blood loss anemia with a hemoglobin of 6.5 g/dL (65 g/L) (normal reference range, 12.7-17.2 g/dL; 127-172 g/L) and thrombocytopenia with a platelet count of 69 × 10^3^/µL (69 × 10^9^/L) (normal reference range, 150-400 × 10^3^/µL; 150-400 × 10^9^/L) that were managed to resolution. Etiology of the thrombocytopenia was believed to be consumptive given large volume losses during surgery, and as heparin-induced thrombocytopenia was ruled out with negative immunoglobulin G testing. Postoperative medication included hydrocortisone 15 mg every morning, hydrocortisone 5 mg in the afternoon, and fludrocortisone 50 μg daily, along with resuming levothyroxine. At his follow-up evaluation 2 weeks after his surgery, he reported having some lower back pain but otherwise felt well with a 40-pound reduction in weight. Given hyponatremia and hyperkalemia (Table 2), his fludrocortisone was increased to 100 μg daily with improvement in sodium up to 136 milliequivalents/liter (mEq/L) (136 mmol/L) (normal reference range, 134-144 mEq/L; 134-144 mmol/L) and potassium down to 4.4 mEq/L (4.4 mmol/L) (normal reference range, 3.5-5.2 mEq/L; 3.5-5.2 mmol/L). At this point, testosterone cypionate was restarted. During his last follow-up, 6 months after surgery, the patient noted fatigue and back pain but otherwise had no concerns.

**Table 2.** Pre- and post-adrenalectomy laboratory data (serum values)

Laboratory markers	Preoperative values (SI units)	Postoperative values (SI units)	Reference range (SI units)
Sodium	138 mEq/L (138 mmol/L)	**132 mEq/L (132 mmol/L)**	134-144 mEq/L (134-144 mmol/L)
Potassium	4.2 mEq/L (4.2 mmol/L)	**5.6 mEq/L (5.6 mmol/L)**	3.5-5.2 mEq/L (3.5-5.2 mmol/L)
Chloride	103 mEq/L (103 mmol/L)	96 mEq/L (96 mmol/L)	96-106 mEq/L (96-106 mmol/L)
Carbon dioxide (CO_2_)	27 mEq/L (27 mmol/L)	21 mEq/L (21 mmol/L)	20-29 mEq/L (20-29 mmol/L)
Blood urea nitrogen (BUN)	18 mg/dL (6.4 mmol/L)	15 mg/dL (5.4 mmol/L)	6-24 mg/dL (2.1-8.6 mmol/L)
Creatinine	1.00 mg/dL (88.4 µmol/L)	1.05 mg/dL (93 µmol/L)	0.76-1.27 mg/dL (67-112 µmol/L)
Glucose	90 mg/dL (5.0 mmol/L)	**117 mg/dL (6.5 mmol/L)**	70-99 mg/dL (3.9-5.5 mmol/L)
Calcium	9.1 mg/dL (2.3 mmol/L)	9.1 mg/dL (2.3 mmol/L)	8.7-10.2 mg/dL (2.2-2.6 mmol/L)
Estimated glomerular filtration rate (eGFR)	>90 mL/min/1.73m^2^	87 mL/min/1.73m^2^	>59 mL/min/1.73m^2^

Abnormal values bolded.

## Discussion

Adrenal masses are frequently discovered incidentally in patients without significant endocrine history [12]. However, in adults with CAH, CT imaging can often reveal adrenal masses, especially among those on inconsistent pharmacological therapy [12]. There have been reported instances of giant bilateral myelolipomas in literature, but it is rare to find such sizes bilaterally. Cases include a 39-year-old man who presented with a 30-cm left adrenal myelolipoma and a 25-cm right adrenal myelolipoma [5], a 51-year-old man with a 34-cm left adrenal myelolipoma and a 20-cm right adrenal myelolipoma [7], a 42-year-old woman with a 30-cm right adrenal myelolipoma and a 25-cm left adrenal myelolipoma [13], a 49-year-old man with a 30-cm left adrenal myelolipoma and a 8-cm right adrenal myelolipoma [9], and a 34-year-old female with a 24-cm left adrenal myelolipoma and a 16-cm right adrenal myelolipoma [14].

A notable feature of this case was the diagnostic uncertainty. The CT characteristics of the masses overwhelmingly suggested that they were benign: the negative density of adipose tissue within the masses, a heterogeneous appearance suggestive of hematopoietic elements, and the bilaterality of the masses [15]. Despite this evidence, the multiple areas of fat density on imaging, along with nonspecific enhancement of soft tissue elements, meant that a liposarcoma could not be ruled out. The European Society of Endocrinology's clinical practice guidelines for incidental adrenal masses (incidentalomas) in 2023 recommend against adrenal biopsy during workup unless there is a history of extra-adrenal malignancy [16]. In the patient presented here, biopsy ultimately did not reveal histological evidence of liposarcoma. This brings forward concern over whether a biopsy is a necessary risk given that clinical features pointed toward an almost certain benign nature of the masses.

Clinical guidelines for management of CAH due to 21-hydroxylase deficiency from 2018 state that “in patients with congenital adrenal hyperplasia, we suggest that bilateral adrenalectomy not be performed” [17]. The reasoning behind this recommendation includes morbidity and mortality related to the surgery, deficiency of beneficial adrenal hormones, and risk of adrenal crisis. Notably, the guidelines report that there are insufficient data for standardized routine screening for adrenal masses, leaving surgical decisions up to clinical judgment [17]. The American Association of Endocrine Surgery's guidelines for adrenalectomy discuss increased risk of adrenal metastases from an adrenal incidentaloma in patients with a history of extra-adrenal malignancy [18]. They recommend definitive resection in cases where there is indeterminate imaging, mass effect from symptomatic tumors, substantive growth or hemorrhage [18]. In patients with imaging characteristics conclusively indicating myelolipoma, resection should only be performed when there are symptoms from mass effect [18]. A meta-analysis published in 2018 looked at indications and outcomes for bilateral adrenalectomy in 48 patients with CAH [19]. The most common indication was having hyperandrogenism symptoms [19]. They found that bilateral adrenalectomy can prove to be an effective therapy when coupled with long-term monitoring.

With increasing size in the retroperitoneal space, opting to monitor giant adrenal myelolipomas carries the risk of spontaneous hemorrhage and hemodynamic instability [11]. On initial presentation, it was evident that the patient had not properly adhered to medication in the past for his CAH. This became part of the discussion, with emphasis placed on the vital nature adhering to medications would have on survival, especially post-adrenalectomy. Close follow-up has helped in ensuring clinical success postoperatively. Shared decision-making with patients is vital to tailoring management to each individual. With a stable clinical state, initially conducting periodic CT scans to assess for progression was an appropriate course of action for the patient presented here. As myelolipomas present as benign lesions, and our patient was presenting with minimal symptoms, we could have clinically monitored without intervention for a longer period. However, given concerns surrounding mass effect and hemorrhage risk, the patient ultimately opted for adrenalectomy, with a positive outcome since the procedure, representing an effective and safe personalized treatment approach.

## Learning Points

- There have been reported instances of giant bilateral myelolipomas in literature, including in patients with uncontrolled CAH; however, bilateral masses both measuring up to 30 cm have rarely been reported.
- Managing giant adrenal masses involves balancing the risks of surgical intervention with potential benefits. Since there is no definitive answer as to whether adrenalectomy or surveillance is the best management of adrenal myelolipomas, quality of life considerations drive decision-making.
- Management of similar benign lesions can consist of periodic imaging, monitoring for clinical risk, and shared decision-making, ensuring that interventions are tailored to each patient.

## Data Availability

Data sharing does not apply to this article as no datasets were generated or analyzed during the current study.
